# Procalcitonin for infections in the first week after pediatric liver transplantation

**DOI:** 10.1186/s12879-017-2234-y

**Published:** 2017-02-15

**Authors:** Vladimir L. Cousin, Kalinka Lambert, Shahar Trabelsi, Annick Galetto-Lacour, Klara M. Posfay-Barbe, Barbara E. Wildhaber, Valérie A. McLin

**Affiliations:** 1 Pediatric Gastroenterology Unit, University Hospitals Geneva, Rue Willy-Donzé 6, 1211 Geneva, Switzerland; 2 Faculty of Medicine, Geneva, Switzerland; 3 Pediatric Emergency Division, University Hospitals Geneva, Geneva, Switzerland; 4 Pediatric Infectious Disease Unit, Department of Pediatrics, University Hospitals Geneva & University of Geneva, Geneva, Switzerland; 5 University Center of Pediatric Surgery of Western Switzerland, Division of Pediatric Surgery, University Hospitals of Geneva, Geneva, Switzerland

**Keywords:** Pediatric, Children, Transplantation, Liver, Infection, Procalcitonin

## Abstract

**Background:**

Procalcitonin (PCT) has become a commonly used serum inflammatory marker. Our aim was to describe the kinetics and usefulness of serial post-operative PCT measurements to detect bacterial infection in a cohort of children immediately after pediatric liver transplantation (pLT).

**Methods:**

We performed a retrospective chart review of a cohort of pLT recipients with serial serum PCT measurements in the first week following pLT. The presence of infection was determined on clinical and biological parameters. Normal PCT was defined as < 0.5 (ng/ml).

**Results:**

Thirty-nine patients underwent 41 pLT. PCT was measured daily during the first week post pLT. Values first increased following surgery and then decreased, nearing 0.5 ng/ml at day seven. Peak PCT reached a median of 5.61 ng/ml (IQR 3.83-10.8). Seventeen patients were considered to have an infection. There was no significant difference in daily PCT or peak PCT between infected and non infected patients during the first post-operative week. AUC of ROC curve for PCT during first week was never higher than 0.6.

**Conclusions:**

We conclude that serial PCT measurements during the first week after pLT is not useful to identify patients with bacterial infections. Rather, we propose that serum PCT may be useful after the first week post pLT.

**Electronic supplementary material:**

The online version of this article (doi:10.1186/s12879-017-2234-y) contains supplementary material, which is available to authorized users.

## Background

Procalcitonin (PCT) is an acute phase protein used especially for prediction of bacterial infection. It was first introduced as a biomarker in the early 90s, and since then, its role as a useful inflammatory marker has been confirmed [1–4]. It is produced by the liver, spleen, lung, adrenal glands, monocytes and macrophages [1, 2, 5]. The classic cut-off for PCT in serum is 0.5 ng/ml. Its serum levels can increase significantly during bacterial infections and after surgery [6, 7]. In the latter case, PCT levels decrease quickly during the first days, but levels may take two weeks to return to baseline [2]. Interestingly, serum PCT does not rise in viral infections or acute cellular rejection following pediatric liver transplantation (pLT) [1, 8–10].

The liver is a major source of PCT. Therefore, predictably, any hepatobiliary surgery will lead to a rise in serum PCT levels [9, 11–14]. However, it is still frequently used as a marker of infection in the early post-operative period after pLT, and, in our center, is drawn once or twice daily per protocol. Data on the utility of PCT early after pediatric liver transplantation (pLT) are sparse and contradictory.

In this retrospective study our primary aim was to describe post-operative variations of PCT in children after a pLT. Our secondary aim was to analyze the value of PCT in distinguishing between patients with and without post-operative bacterial infections in the first seven days following pLT.

## Methods

We performed a single center retrospective chart review of children following pLT between 01/2010 and 04/2015 in our national liver transplantation center. Ethics committee approval and informed consent was obtained, explicitly authorizing chart review studies for publication.

All children had at least one PCT measurement daily during the first week after pLT. We excluded patients without a single procalcitonin value and children for whom a pathogen was identified in the organ preservation solution (*N* = 3). We recorded PCT levels drawn at the same hour each day (morning labs) or PCT at any other time if there was only one level available. We used only one PCT value per day. Day 0 was defined as the day the surgery was completed. The highest PCT value of the week was termed ‘peak PCT’. PCT was measured by electro-chemiluminescence immunoassay (Roche Diagnostic AG, Rotkreuz, Switzerland) and expressed in nanograms per milliliter (ng/mL). We compared PCT data to C-reactive protein (CRP) levels. CRP was measured by immunoturbidimetry (Roche Diagnostic AG, Rotkreuz, Switzerland). The highest CRP value of the week was termed ‘peak CRP’.

We defined infection as any patient needing either a change of antibiotic or addition of an antifungal during the first week after pLT, based on clinical or laboratory factors including: clinical deterioration, hemodynamic instability, or positive blood- or urine cultures, tracheal aspirates or sputum with or without other clinical signs of infection.

We collected demographic and surgical data from patient records to analyze their potential impact on infection occurrence or their relationship to PCT level in the post-op period. Diagnoses were classified as follows: biliary atresia, other cholestatic (PFIC, Alagille syndrome, secondary biliary cirrhosis), acute liver failure, metabolic disease, immunological disease (overlap syndrome, sclerosing cholangitis), tumor and other (cystic fibrosis, cirrhosis of unknown origin). Graft types were separated into whole liver and technical variant for split and reduced liver.

### Statistical analysis

For numerical variables, a Student *T*-test or a Wilcoxon test was used depending on variable distribution. A Chi-square test or a Fisher’s exact test were used to compare categorical variables. Linear regression was used to find impact of factor on peak PCT or PCT at 4^th^ day. P-values inferior to 0.05 were considered significant. Statistical analyses were carried out with STATA software, version 13.0 (StataCorp, College Station, TX, USA).

## Results

Thirty-nine patients underwent 41 pLT. Two patients received 2 pLT more than 6 months apart; these were counted as separate events. One patient died 2 months after pLT from multiple organ failure. The median age at pLT was 37 months (IQR 10-144) with a median weight of 13.6 kgs (IQR8.4-40).

All patients received a liver from a deceased donor, either whole or a technical variant. Immunosuppression for all patient included basiliximab induction followed by tacrolimus as standard calcineurin inhibitor immunosuppression. Steroids were used in all but 4 patients who were included in the ChilSFree study (http://www.espghan.org/about-espghan/research/immunomonitoring-after-paediatric-liver-transplantation-in-search-for-markers-of-over-or-under-immunosuppression/). Patients having received chemotherapy prior to transplant did not receive basiliximab induction. All patients received broad spectrum antibiotics intra-operatively and for 5 days following surgery.

### PCT kinetics following pLT

**Fig. 1:**
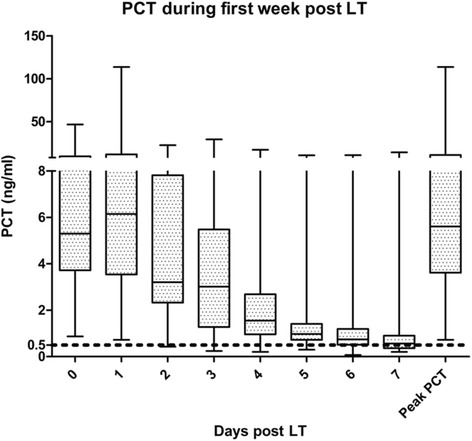
Daily and peak PCT values during the first week post liver transplantation. The median is indicated with a horizontal mark for each day. PCT is expressed in nanogram per milliliter. PCT: procalcitonin

Daily post-transplant serum PCT values and peak PCT are summarized in Fig. 1. Serum levels rised during post operative days 0 and 1. Subsequently, PCT decreased to approach the laboratory cut off of 0.5 ng/ml after seven days. Only one patient had a PCT level below the limit prior to the third post operative day.

### PCT in patients treated for a bacterial infection

**Fig. 2:**
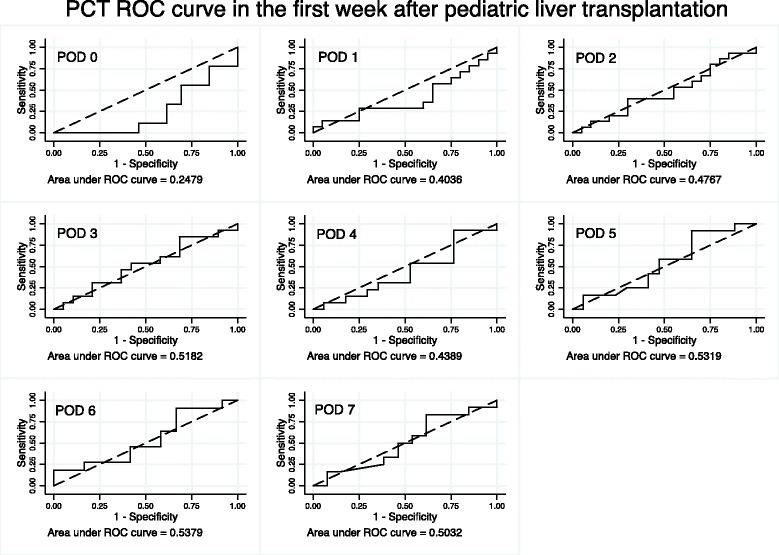
ROC curve of PCT during the first week after the transplantation. AUC: area under the curve. PCT: procalcitonin. POD: post operative day

**Fig. 3:**
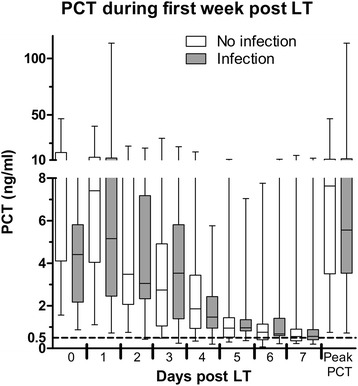
Mean daily and peak PCT values for the groups with and without infection. PCT: procalcitonin. PCT is expressed in nanograms per milliliter. PCT: procalcitonin. ALF: acute liver failure

During the first seven days following pLT, seventeen transplanted patients were clinically considered to have a bacterial infection while 24 were not. Of the seventeen patients, 6 had clinical signs of infections: 3 showed signs suggestive of septic shock, 2 displayed a general worsening and 1 had clinical and radiological evidence of pneumonia. For the 11 others, all presented at least one positive culture: 2 blood cultures, 4 urine cultures, 2 tracheal aspirates and 3 intra-abdominal samples collected during transplant surgery. Most also presented with fever and one was in septic shock immediately preceding transplantation. *Pseudomonas aeruginosa* was the most frequently identified pathogen (3/17). The following were each identified in one patient: *Staphylococcus epidermidis*, *methicillin-sensible Staphylococcus aureus*, *Streptococcus parasanguinis*, *Enterococcus faecalis*, *Escherichia Coli*, *Enterobacter cloacae*, *Klebsiella pneumonia* and *oxytoca*, *Proteus mirabilis*, *Stenotrophomonas maltophilia* and *Candida tropicalis*, (Additional file 1: Table S1). As shown in table 1, there were no significant differences in demographic or surgical variables between patients with and without an infection. Figure 2 illustrates the area under the curve for daily PCT during the first week post LT, which did not reach statistical significance. Figure 3 compares serum PCT levels between the two groups. There were no significant differences in daily serum PCT measurements or in peak PCT. In both groups, serum values returned to normal between post operative days 5 and 6.

**Table 1:** Population characteristics for patients with infection (n = 17) and without infection (n = 24). Age, weight and peak serum PCT levels are represented as median with interquartile range. Ischemia times are represented as mean with 95% confidence interval. pLT: pediatric liver transplantation. ALF: acute liver failure. CI: confidence interval. IQR: interquartile range

	No Infection N = 24 (%)	Infection N = 17 (%)	*P* value
DIAGNOSIS			
Biliary atresia	12 (50)	6 (35.3)	0.35
Other cholestatic	1 (4.2)	1 (5.9)	1
ALF	3 (12.5)	1 (5.9)	0.60
Metabolic	3 (12.5)	1 (5.9)	0.60
Immunologic	1 (4.2)	2 (11.8)	0.56
Tumor	3 (12.5)	1 (5.9)	0.60
Other	1 (4.2)	5 (29.4)	0.06
Male	15 (62.5)	9 (52.9)	0.50
Female	9 (37.5)	8 (47.1)	0.50
Age at pLT (months)	26 (IQR 9-131)	45 (IQR 13-149)	0.80
Weight (kgs)	13.3 (IQR 7.6-38)	17 (IQR 8.5-40)	0.80
SURGICAL			
Whole	10 (41.7)	10 (58.8)	0.27
Technical variant	14 (58.3)	7 (41.2)	0.27
Total ischemia time (min)	378 (CI 346-409)	416 (CI 355-476)	0.30
Warm ischemia time (min)	61 (CI 51-70)	54 (CI 43-63)	0.24
Peak PCT (ng/ml)	7.63 (IQR 3.6-10.3)	5.6 (IQR 3.9-10.8)	0.60

By way of comparison, we also looked at CRP measurements in both patient groups (Additional file 2: Figure S1). Except for day 6, there were no statistical differences in daily CRP values or peak CRP between the groups with and without infection. In both groups, we observed an initial rise followed by a steady decline during the first week after pLT.

### Recipient and surgical variables and PCT

We analyzed the following recipient and surgical variables for their potential association with increased serum PCT in the early days following pLT: age or weight at transplantation, diagnosis, and surgical- ischemia time (warm, cold and total) and graft type. However, none of these correlated with serum PCT levels at day 4 or peak PCT, although there was a trend for patients having received a technical variant (*p =* 0.052) to have a lower peak PCT levels. We chose POD 4, because in the literature it is the earliest timepoint at which PCT has been reported to be of some value after pLT [8].

One of the major differences between whole and split grafts is the presence of a cut surface and thus a prolonged handling/dissection of the very organ that produces PCT. We therefore performed a *post-hoc* analysis to compare patients with a whole liver to those with a partial liver. Patients receiving partial grafts (technical variants) were younger and lighter (*p* < 0.001). They also had a significantly shorter total ischemia time (*p* = 0.02) (Additional file 3: Table S2). Moreover, PCT values were significantly different until the fifth post-operative day according to transplantation type: day 1 *p =* 0.02, day 2 *p =* 0.009, day 3 *p =* 0.02, day 4 *p =* 0.03. On day 5, the p value reached 0.47 and did not differ between graft type thereafter. Peak PCT was also significantly higher in whole graft recipients (*p =* 0.05).

## Discussion

### Serum PCT does not detect bacterial infection early post pLT

In this study, we analyzed the kinetics of PCT during the first week after pLT. In all patients serum PCT rose during post-operative days 0 and 1 and returned to baseline by the end of the first week. The general kinetics of post-operative PCT were similar to other reports [9, 12, 15, 16]. After an initial increase, PCT decreased to its baseline level at a different rate for each patient. In a report on PCT kinetics in lung transplantation, only 50% of patients had a PCT < 0.5 ng/ml after one week [17].

We also studied whether serum PCT levels were of value in distinguishing pLT recipients with and without early post-operative bacterial infections. In total, the two groups did not differ in PCT kinetics or peak level in the early post operative period, confirming the findings of others [9, 12, 15]. We observed from our retrospective chart review that PCT levels were seldom used in the clinical decision to treat suspected infection. Rather, clinical signs were the main criteria to guide decision-making. In a recent report by Mandell et al, who also used clinical criteria for infections in pediatric intensive care, the authors concluded that PCT measurements did not predict bacterial infections reliably enough to be clinically useful [18].

The liver has a crucial role in PCT regulation, and therefore it follows that major hepatic surgery can contribute to upsetting its homeostasis [11, 14, 19]. Thus, it is reasonable to assume that early post pLT perturbations in serum PCT levels are both the product of the surgery and of the immunological storm created by the allograft. Together, these processes overshadow the response of the new allograft (and of other cells that produce PCT) to an infectious stimulus [14, 20]. The magnitude of the immune stimulus is such that even in hematopoietic stem cell transplantation, in which the liver is in its native state, PCT seems to be a suboptimal marker of infection [19].

Other groups proposed that PCT values were not of use for the diagnosis of infection before POD 4 [8, 21, 22]. We would like to extend the same recommendation by suggesting that PCT is of little value prior to POD 5. We proposed day 5 because PCT started to diminish at this time, and there was no difference between graft type starting on POD 5. Thus, prior to POD 5, it is imperative to consider serum levels with clinical findings, while bearing in mind that patients with whole grafts may display higher serum values. Moreover, it is essential to keep in mind that early after the procedure a normal level does not rule out infection, something of importance if antimicrobial regimen is being weaned.

In this small cohort, partial grafts were associated with a lower peak PCT. The reasons for this novel finding are unclear. One possibility is that technical variant grafts had a significantly shorter total ischemia time than whole organs, thereby impacting serum PCT values known to rise in ischemia [14, 16, 19, 22–24]. On the other hand, ischemia time was not significantly associated with PCT in the linear regression, suggesting that ischemia time alone does not solely explain the difference. More likely is the fact that small grafts were implanted into smaller children who received proportionately more intra- and post- operative fluids compared to older children thereby leading to a relative dilution of measurable PCT until day 5.

Other reports have suggested that recipients of grafts from living donors displayed lower PCT levels than those receiving grafts from deceased donors [16, 22]. Ischemia time may have contributed to this difference [16]. Even if we could not account for it, it is important to remember that donor history has been shown to contribute significantly to PCT levels [25]. Moreover, donor PCT was shown to predict early cardiac graft failure [26, 27], something which we could not verify in the present study.

CRP is an acute phase protein; its level has been reported to have a high sensitivity but low specificity to detect inflammation triggered by surgery, transplantation or numerous post-pLT complications [12, 28–30]. It has been shown that CRP offers no advantage over PCT to diagnose infection following liver transplantation [9]. Yet, unlike PCT, peak CRP may help predict infection in the setting of pLT [15]. Therefore, we analyzed CRP values, but there was no difference between groups with and without a clinically-suspected infection except on day 6.

In light of our findings, we performed a simple *post-hoc* cost-benefit analysis. Most patients in our cohort had at least 2 PCT levels drawn daily per protocol during the first week post LT. Considering that every PCT level drawn before POD 5 was not useful, this amounted to 10 PCT measurements per transplantation totaling 410 unnecessary PCT measurements in our cohort amounting to a total USD 34000 for the length of the study. In other words, savings would amount to 840 USD per transplant. Similarly, non-contributory daily CRP measurements totaled USD 4000. In total, combined with CRP costs, we could have saved in excess of USD 38’000 by cutting back on daily measurements of PCT and CRP during early post-transplant follow-up, something we have now implemented.

### Limitations of the study

Our study was marred by several limitations. First, the sample size was small, owing to the recent use of PCT in our institution. Further, we did not have a daily PCT value for all patients. Next, the definition of severe bacterial infection was clinical and not based on extensive bacteriological work up and proof. However, it was representative of the clinical practice in a post-transplant intensive care unit with acutely ill patients, under immunosuppressive drugs. Moreover, this definition is also used in other reports on PCT use in pediatric intensive care units [18].

## Conclusion

In our small but representative cohort we have shown that PCT was of little value in distinguishing patients with or without severe bacterial infection in the first week post pLT. Rather, it is possibly a valuable diagnostic tool beyond the first post-operative week because it regains its normal value at the end of the first week. We recommend the limited and cautious use of this costly test in the first week follow up of post pLT patients.

## Additional files

Additional file 1: Table S1. Description of bacteria found and antibiotic used in infected patients. Patient with only clinical infection were noted as “Clinical”. “→” mean “replace by”. “+” mean “adjunction”. (DOCX 72 kb)
Additional file 2: Figure S1. Representation of CRP during the first week after pLT. * *p* < 0.05. CRP is expressed in milligram per liter. CRP: C reactive protein. (JPG 1339 kb)
Additional file 3: Table S2. Comparison between patients with a whole liver or a partial liver graft. Significant p values are in bold. PCT: procalcitonin. pLT: pediatric liver transplantation. (DOCX 64 kb)
