# Supplementary material for: Procalcitonin for infections in the first week after pediatric liver transplantation
Source: BMC Infect Dis. 2017 Feb 15;17:149. doi: 10.1186/s12879-017-2234-y (PMC5311857; doi:10.1186/s12879-017-2234-y)
Supplement: Additional file 1: Table S1. — Description of bacteria found and antibiotic used in infected patients. Patient with only clinical infection were noted as “Clinical”. “→” mean “replace by”. “+” mean “adjunction”. (DOCX 72 kb) [file 12879_2017_2234_MOESM1_ESM.docx]

Table S1:

|  | **Bacteria** | **Antibiotic** |
| --- | --- | --- |
| 1 | Stenotrophomonas maltophilia | Trimethoprim-sulfamethoxazole |
| 2 | Clinical | Ceftriaxone |
| 3 | Staphylococcus epidermidis | Piperacillin-tazobactam + vancomycin |
| 4 | Enterobacter cloacae & Klebsiella pneumoniae | Piperacillin-tazobactam + vancomycin |
| 5 | Proteus mirabilis | Ceftriaxone + metronidazol --> co-amoxicillin |
| 6 | Clinical | Meropenem + voriconazole + teicoplanin |
| 7 | Pseudomonas aeruginosa | Meropenem + gentamicin |
| 8 | Klebesiella oxytoca | Meropenem |
| 9 | Escherichia Coli & Enteroccocus faecalis | Piperacillin-tazobactam |
| 10 | Candida tropicalis | Amphotericin B |
| 11 | Pseudomonas aeruginosa | Piperacillin-tazobactam + gentamicin + i.colistin |
| 12 | Pseudomonas aeruginosa | Imipenem--> ceftazidime + trimethoprim/sulfamethoxazole + levofloxacine + i.collistine |
| 13 | Clinical | Meropenem + vancomycin + amphotericin B + amikacin |
| 14 | Clinical | Meropenem + vancomycin |
| 15 | Clinical | Piperacillin-tazobactam--> meropenem + teicoplanin |
| 16 | Methicillin-sensitive Staphylococcus aureus & Streptococcus parasanguinis | Cefuroxime --> piperacillin-tazobactam + vancomycin -->co-amoxicillin |
| 17 | Clinical | Meropenem + vancomycin |
