# Supplementary figures and images for: Procalcitonin for infections in the first week after pediatric liver transplantation
Source: BMC Infect Dis. 2017 Feb 15;17:149. doi: 10.1186/s12879-017-2234-y (PMC5311857; doi:10.1186/s12879-017-2234-y)

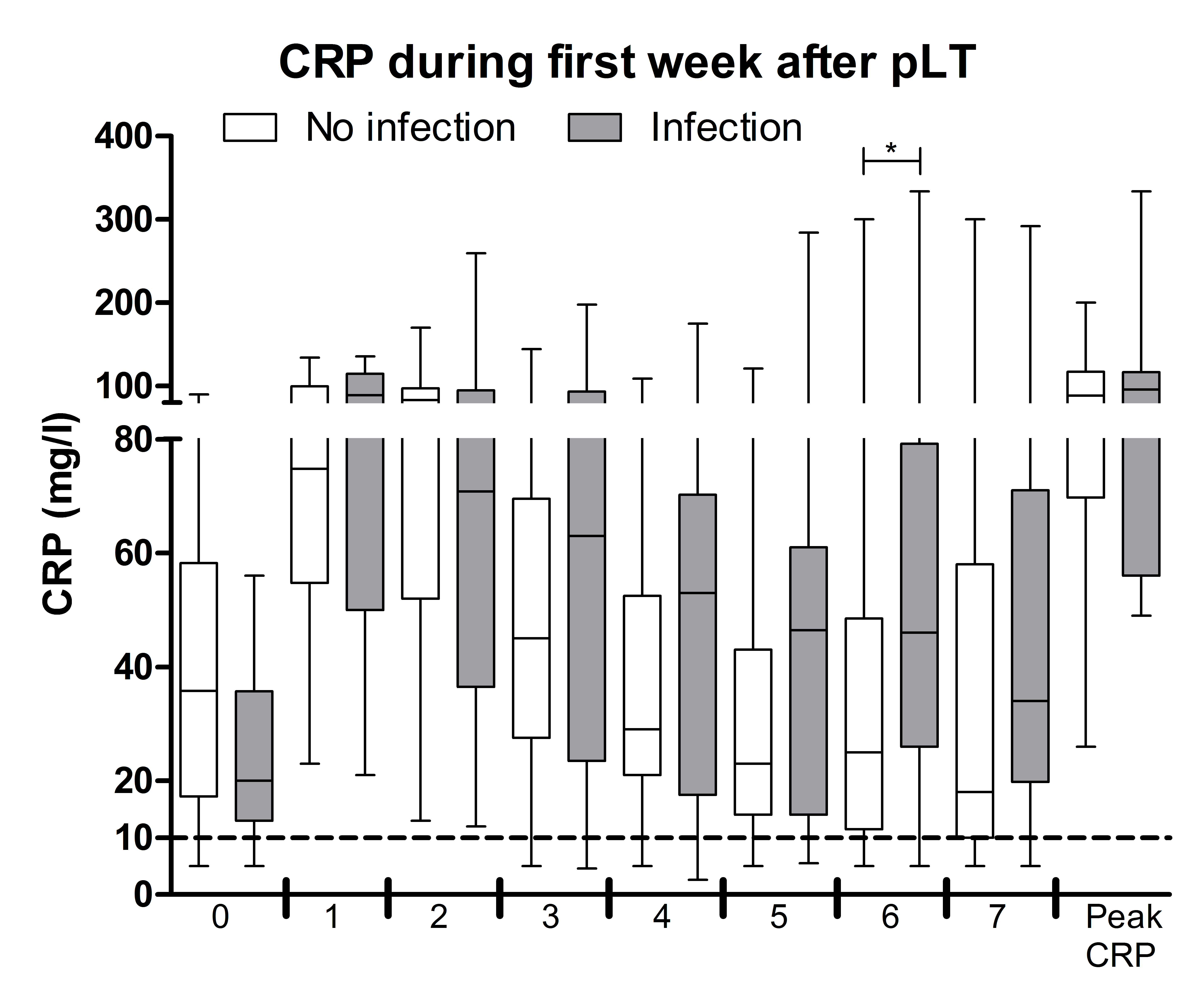

Supplement: Additional file 2: Figure S1. — Representation of CRP during the first week after pLT. * p < 0.05. CRP is expressed in milligram per liter. CRP: C reactive protein. (JPG 1339 kb) [file 12879_2017_2234_MOESM2_ESM.jpg]
