# Supplementary material for: Procalcitonin for infections in the first week after pediatric liver transplantation
Source: BMC Infect Dis. 2017 Feb 15;17:149. doi: 10.1186/s12879-017-2234-y (PMC5311857; doi:10.1186/s12879-017-2234-y)
Supplement: Additional file 3: Table S2. — Comparison between patients with a whole liver or a partial liver graft. Significant p values are in bold. PCT: procalcitonin. pLT: pediatric liver transplantation. (DOCX 64 kb) [file 12879_2017_2234_MOESM3_ESM.docx]

Table S2:

|  | **Whole liver (n=20)** | **Partial Liver (n=21)** | **p value** |
| --- | --- | --- | --- |
| **Age at pLT (months)** | 139.5 (33-176.5) | 13 (9-37) | **<0.001** |
| **Weight at pLT (kgs)** | 35.5 (13.5-46.5) | 9 (6.8-13.6) | **<0.001** |
|  |  |  |  |
| **Total ischemia time (min)** | 430.5 (359-472.5) | 366 (290-429) | **0.02** |
| **Warm ischemia time (min)** | 50.5 (46.5-55.5) | 59 (45-65) | 0.3 |
| **Cold ischemia time (min)** | 366.5 (313.5-417.5) | 303 (233-370) | **0.02** |
|  |  |  |  |
| **PCT day 0 (ng/ml)** | 5.81 (4.04-9.89) | 4.66 (1.6-8.03) | 0.22 |
| **PCT day 1 (ng/ml)** | 8.02 (4.99-17.04) | 4.81 (1.8-7.57) | **0.02** |
| **PCT day 2 (ng/ml)** | 4.83 (2.97-11.9) | 2.9 (0.98-3.81) | **0.009** |
| **PCT day 3 (ng/ml)** | 3.7 (1.63-8.79) | 1.42 (0.74-3.58) | **0.02** |
| **PCT day 4 (ng/ml)** | 2.17 (1.23-3.83) | 1.13 (0.69-1.86) | **0.03** |
| **PCT day 5 (ng/ml)** | 1 (0.85-1.42) | 0.93 (0.58-1.41) | 0.47 |
| **PCT day 6 (ng/ml)** | 0.66 (0.42-1.19) | 0.79 (0.61-1.48) | 0.39 |
| **PCT day 7 (ng/ml)** | 0.43 (0.36-0.87) | 0.61 (0.5-0.9) | 0.37 |
| **Peak PCT (ng/ml)** | 7.36 (4.44-22.4) | 5.56 (1.81-8.53) | **0.05** |
|  |  |  |  |
| **Infection** | 10 (50%) | 7 (33%) | 0.5 |
